# Pityriasis rubra pilaris after COVID-19 vaccination: successful treatment with ustekinumab^⋆^

**DOI:** 10.1016/j.abd.2023.07.009

**Published:** 2024-04-22

**Authors:** Bárbara Vieira Granja, Patrícia Amoedo, Nuno Preto Gomes, Catarina Costa, Filomena Azevedo, Sofia Magina

**Affiliations:** a Department of Dermatology and Venereology, Centro Hospitalar Universitário de São João, EPE, Porto, Portugal; b Department of Pathology, Centro Hospitalar Universitário de São João, EPE, Porto, Portugal; c Department of Biomedicine, Faculty of Medicine, Universidade do Porto, Porto, Portugal

Dear Editor,

Pityriasis Rubra Pilaris (PRP) is a rare erythematous papulosquamous inflammatory dermatosis.^1^

Since the approval of mRNA vaccines for COVID-19, the dermatology community strived to characterize the adverse cutaneous effects associated with their administration.

We report a case of PRP following administration of mRNA Pfizer-BioNTech COVID-19 vaccine with refractoriness to first-line systemic therapy that was successfully treated with ustekinumab.

A 69-year-old Caucasian woman, with no relevant medical history, was referred due to a 6-month course of generalized rash that did not improve with highly potent topical and systemic corticosteroids (1 mg/kg/day) or oral cyclosporin (4 mg/kg/day). She reported a facial erythematous scaly rash 2-days after receiving the first dose of the Pfizer-BioNTech COVID-19 vaccine. A few days following the second vaccine dose the patient noticed a worsening of the rash with progression to the trunk and limbs. She reported intense itch and denied a personal or family history of skin diseases or recent infections. On physical examination, she presented with sharply demarcated confluent orange-red squamous plaques extending from her scalp to the arms and proximal thighs with islands of sparing. There were scattered erythematous plaques over the lower legs and orange-red waxy palmoplantar keratoderma (Fig. 1). Blood tests showed leukopenia (3150 mm^3^) with normal eosinophil counts. Serological testing for the virus and extended immunological study were negative. Computed tomography was unremarkable. Histological examination revealed moderate acanthosis, with a few broad rete ridges, hyperkeratosis, and parakeratosis with mild spongiosis (Fig. 2). In the superficial dermis, there was a mild perivascular chronic inflammatory cell infiltrate. Genetic testing was negative for CARD14 mutations.

**Figure 1:**
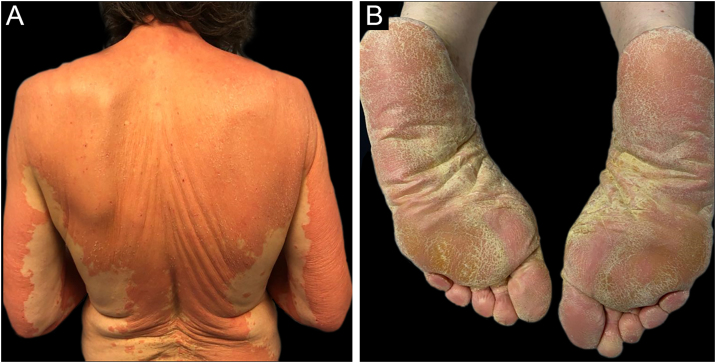
Orange-red squamous plaques with islands of sparing in the trunk and both arms (A). Orange-red waxy and symmetrical palmoplantar keratoderma (B).

**Figure 2:**
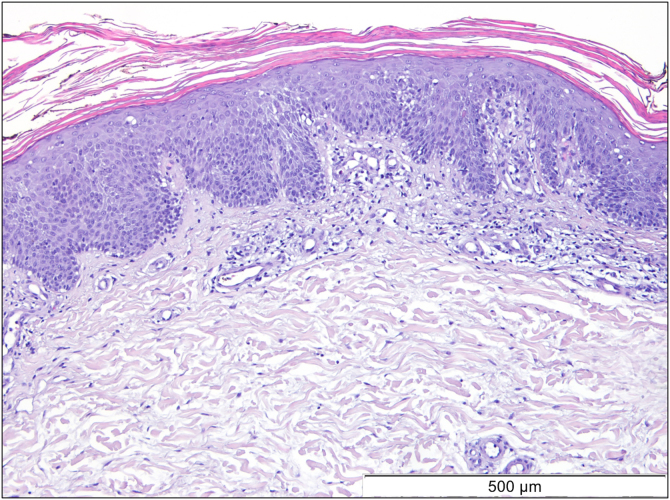
Histological examination revealed moderate acanthosis with hyperkeratosis and parakeratosis: mild epidermal spongiosis and, in the superficial dermis, a mild perivascular chronic inflammatory cell infiltrate (Hematoxylin & eosin).

She was initially treated with Methotrexate (MTX) 12.5 mg once a week for 4 weeks, however, it was necessary to increase it to 15 mg during 12 weeks without any improvement. Hands and feet topical PUVA (Psoralen and Ultraviolet A therapy) was performed concomitantly to MTX with no additional benefit. Subsequently, acitretin 25 mg every other day was prescribed but the patient stopped it 15 weeks later due to an unsatisfactory response and increase in transaminases (10 times de upper limit of normal) which then normalized after the drug withdrawal. As the patient showed refractoriness to first-line systemic therapies we decided to start therapy with ustekinumab (45 mg at week 0, week 4 and then every 12 weeks). At week-4 of ustekinumab almost complete response was achieved, only residual erythema of the face was observed (Fig. 3). The response was maintained during 6 months of follow up and no further COVID-19 vaccine was done.

**Figure 3:**
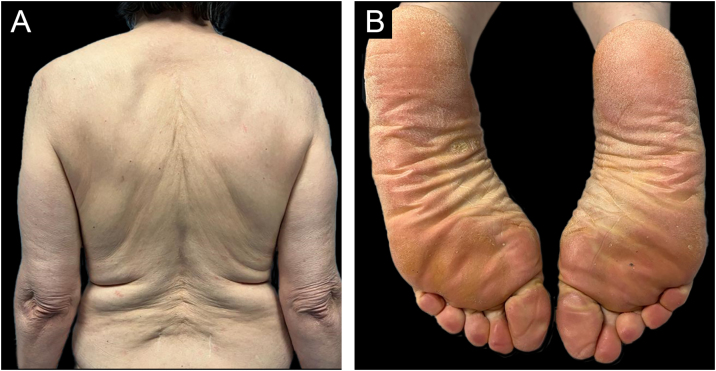
After four weeks of treatment with ustekimumab, there was a great improvement of the lesions (A‒B).

The etiology and pathogenesis of PRP remains unclear, most cases are sporadic although infections, drugs, malignancies and vaccines are all possible triggers.^1^ Since the approval of SARS-CoV-2 vaccines a crescent number of COVID-19 vaccine-induced PRP cases have been reported.^2^, ^3^ Given the existing literature, the fact that PRP is a rare condition and the close temporal relationship with COVID-19 vaccination we suggest a causal relationship in the present case.

Treatment of PRP is challenging due to lack of evidence and the gold standard of treatment are oral retinoids followed by methotrexate.^1^ In most COVID-19 vaccine-induced PRP cases treatment was systemic, including retinoids, corticosteroids and methotrexate.^2^, ^3^ More recently, for patients with failure or contraindication for conventional therapies, biologics have emerged as a promising alternative.^2^, ^4^ Those treatments were initially mostly empirical, however Feldmeyer et al. identified higher expression levels of pro-inflammatory innate cytokines and adaptative T-cell cytokines (especially the Th17 cytokines IL-17 and IL-22) in lesional skin of PRP, suggesting the role of IL-23-Th17-pathway in the disease pathogenesis.^5^ In fact, interleukin 17 (IL-17), IL-23, and IL-12/23 antagonists have been used with success.^2^, ^3^, ^4^, ^6^

Our patient’s response to ustekinumab after failure of conventional therapies provides additional evidence for the role of IL-23-Th17-pathway in the disease pathogenesis and effectiveness of anti-IL12/23 agents.

## Financial support

None declared.

## Authors’ contributions

Bárbara Vieira Granja: The study concept and design; writing of the manuscript or critical review of important intellectual content; critical review of the literature; final approval of the final version of the manuscript.

Patrícia Amoedo: The study concept and design; writing of the manuscript or critical review of important intellectual content; final approval of the final version of the manuscript.

Nuno Preto Gomes: Writing of the manuscript or critical review of important intellectual content; final approval of the final version of the manuscript.

Catarina Costa: Critical review of important intellectual content; final approval of the final version of the manuscript.

Filomena Azevedo: Intellectual participation in the propaedeutic and/or therapeutic conduct of the studied cases; critical review of the literature; final approval of the final version of the manuscript.

Sofia Magina: Intellectual participation in the propaedeutic and/or therapeutic conduct of the studied cases; critical review of the literature; final approval of the final version of the manuscript.

## Conflicts of interest

None declared.
